# The Mediating Role of Positive and Negative Affect in the Association of Perceptions of Parenting Styles with Resilience among Adolescents with Addicted Parents

**DOI:** 10.18502/ijps.v15i4.4295

**Published:** 2020-10

**Authors:** Zahra Nikmanesh, Noorali Oshtorak, Mehdi Darvish Molla

**Affiliations:** 1 Department of Psychology, School of Education and Psychology, University of Sistan and Baluchestan, Zahedan, Iran.; 2 The Education Organization, Zabol, Iran.

**Keywords:** *Adolescent*, *Addicted Parent*, *Negative Affect*, *Positive Affect*, *Perceived Parenting Styles*, *Resilience*

## Abstract

**Objective:** This study aimed to investigate the mediating role of positive and negative affect in the association of perceptions of parental involvement, autonomy support, and warmth with resilience among Adolescents with Addicted Parents.

**Method** **:** In this descriptive-correlational study, 63 Adolescents of Addicted Parents studying in Zahak and Hirman, located in Sistan and Baluchestan Province in Iran, were selected using convenience sampling method. The participants completed the questionnaires on perceptions of parenting styles, resilience, and positive and negative affect. Data were analyzed via the correlation coefficient and path analysis.

**Results: **The results showed that the adolescents’ perceived parenting styles were directly and significantly related to positive affect and resilience (P < 0.01). Moreover, the results of the path analysis indicated that mediated by positive affect, the adolescents’ perceived parenting styles predicted resilience indirectly (P < 0.01).

**Conclusion: **Given the results of this study, perceived parenting styles can directly and indirectly predict resilience. It can be concluded that adolescents’ perceived parenting styles can enhance resilience among them through promoting positive affect.

Adolescence is a crucial period of life, as many unhealthy behaviors often start in adolescence. Unhealthy behaviors that begin during this period usually persist into adulthood (1). This is while one of the factors that influence the incidence of risky behaviors (eg, drugs abuse) is the presence of an addicted parent in a family (2, 3). Moreover, families with a addicted member experience high levels of domestic violence (4). Family violence leads to behavioral problems in boys and emotional problems in girls (5). Researchers (6) found that emotional and behavioral problems in adolescents were directly related to addiction. Furthermore, addiction of a family member increases the likelihood of drug abuse by other family members (7). 

Resilience is among factors that are inversely correlated with drug abuse. High resilience reduces the likelihood of abusing drugs among adolescents (8). The term resilience can be defined as the ability to recover from or adjust to difficult situations (9). According to Arce, Simmons, Stein, Winkielman, Hitchcock and Paulus (10), high-resilient people are more likely to display positive emotions when dealing with unknown and neutral emotional events. Moreover, resilience prevents the incidence of psychological issues among youths and young adults and protects them from the psychological effects of problematic events (11). Resilient people have high levels of mental health and desirable self-regulation skills and are less likely to involve in risky behaviors (12). 

Accordingly, given the positive results of having high resilience, examining factors that enhance the level of resilience is of particular importance. People with high self-confidence and self-regulation skills and those who are under the active supervision of their parents experience high levels of resilience (13). In addition, the emotion regulation strategies and coping styles are significantly related to resilience (14). Culture is another factor that affects resilience, such that in different cultures, factors affecting the level of resilience may vary (15). Family factors and parenting styles are other factors that play key roles in promoting resilience among adolescents (16). 

A family is among the most important factors which play protective roles against the abuse of drugs among adolescents (17). The authoritative parenting style (controlling children accompanied by establishing loving and warm relations) acts as a protective shield against the abuse of drugs (18). Based on the results of Piko and Balázs (19), the acceptance/involvement of parenting style can positively predict resilience (20). Numerous studies conducted to examine the impacts of parenting styles have indicated that adolescents who have a parent with the authoritarian parenting style are not likely to use illegal drugs (21, 22) and have good levels of academic achievement (23). Furthermore, a family plays an important role in preventing adolescents from abusing drugs (24-26). In the same line, Cleveland, Feinberg, Bontempo, and Greenberg (27) concluded that while peers play a key role in the middle and late adolescence, families play a very significant role in their early adolescence. Studies have demonstrated that parenting styles are associated with self-confidence and hope (28, 29), life satisfaction (30, 31), well-being (32), depression, lower risky behaviors among adolescents (33), and coping styles and strategies (34, 35). Along with these studies, several studies have pointed out the role of culture in parenting styles. In this regard, perceived parenting styles may vary in different cultures and thus be associated with different outcomes (36-38). Wong (39) mentioned the inverse correlations of high perceived parenting involvement and autonomy support with drug abuse. Moreover, the mediating role of self-regulation was mentioned in this study. The perceived parenting styles have 3 dimensions: involvement, autonomy support, and warmth. Parental involvement refers to the amounts of time that parents devote to their children. Parental autonomy support is defined as the degree to which parents value their children’s activities and give them the right to decide. Parental warmth refers to parents’ responsiveness and sensitivity towards their children (40). 

Studies have demonstrated that perceived parenting styles play an important role in regulating emotions and development of emotion regulation in children and adolescents (41, 42). In addition, happiness is another factor that is positively related to perceived parenting styles (43). Furthermore, since positive emotions increase mental flexibility and creativity, they play a significant role in enhancing resilience (44). One of the most important sources of resilience is positive affect (45), which refers to the extent to which an Individual experiences positive feelings, interest, and alertness and it has 3 subdimensions: amiability (eg, being happy, lively, and cheerful), self-esteem (eg, being reliable, strong, and courageous), and alertness (eg, being alert, focused, and determined). Negative affect is characterized by anger, contempt, disgust, guilt, nervousness, and fear (31). 

 Nikmanesh, Baluchi, and Motlagh (46) found that social support and positive affect play effective roles in predicting addition relapse, which means that low positive affect and social support can predict addition relapse. Research has shown that resilience is significantly associated with optimism (47, 48). Moreover, emotion regulation is related to positive and negative affect, such that reappraisal (an emotion regulation strategy) is negatively correlated with negative affect and positively related to positive affect (49). This is while there is a significant positive association between positive affect and resilience (50, 51). However, there is an inverse relationship between resilience and negative affect (51). Taken together, perceived parenting styles may be related to affects, which in turn is related to resilience. However, to our knowledge, no published research has directly examined the mediating role of affects in the relationship between adolescents’ perceived parenting styles and resilience.

Given that, on the one hand, adolescence is a critical period of life and, on the other hand, parents’ addiction affects many aspects of children’s lives and increases the risk of using drugs in adolescents, examining mental features which play key roles in promoting people’s resilience to drug abuse is of significant importance. Indeed, this study sought to investigate the effects of an external factor (family) and an internal factor (positive and negative affect) on resilience among adolescents with addicted parents. Thus, the present study aimed to examine the mediating role of positive and negative affect in the relationship between adolescents’ perceived parenting styles and resilience (figure 1).

## Materials and Methods

***Participants***

This descriptive correlational study included all senior and junior high school students in the academic year of 2013-2014 who had addicted parents and lived in Zahak and Hirman, Sistan and Baluchestan Province in Iran. In path analysis, Jackson (2003) stated that the minimum sample size is determined by the number of model parameters that require statistical estimates (q). An acceptable sample size-to-parameters ratio would be 10: q (52). The sample was selected using the accessible sampling method. Finally, 63 adolescents (14 to 17 years old) with addicted parents were selected as the sample. 

***Data Collection Methods***

The researcher worked as a consultant at high schools in Zahak and Helman. Therefore, through participating in various classes and explaining the main aims of conducting the present study, the researcher asked those students who had addicted parents and were eager to take part in this study to complete the questionnaires. Those students whose parent had taken drugs for at least a year were considered in this study. To observe the ethical considerations, all the questionnaires were completed without mentioning the participants’ names and addresses. Moreover, the participants expressed their consent to take part in this study. To observe the ethical considerations, at first, parental consent was obtained; then, all the questionnaires were completed without mentioning the participants’ names and addresses. 

***Measures***

***The Perceived Parenting Styles Scale***

This scale was developed by Robbins using the self-determination theory (40). The original version of this scale includes 42 items, 21 items for fathers and 21 items for mothers (the items are repeated in 2 halves for mothers and fathers). This scale is scored based on a seven-point Likert-type scale (1 to 7). This scale is designed for those who are in their late adolescence or their early adulthood. This scale has 6 subscales which examine mothers’ autonomy support, mothers’ involvement, and mothers’ warmth, as well as, fathers’ autonomy support, fathers’ involvement, and fathers’ warmth. Involvement refers to the amount of time and resources that parents devote to children. Moreover, it shows the extent to which parents pay positive attention to raising their children and the amount of time they devote to it. Autonomy support refers to the degree to which parents value their children’s activities, encourage them to solve problems, and give them the rights to make their minds and to participate in the process of making decisions. Warmth refers to parents' responsiveness, sensitivity, and respect towards their children. These parents are encouraging, stable, and intimate (40). Reshvanloo and Hejazi (53) reported that the Cronbach’s alpha coefficient of this scale was 0.82 and the coefficients of its subscales ranged from 0.82 to 0.91. In this study, the Cronbach’s alpha coefficient was 0.81.

***The Connor-Davidson Resilience Scale***

To examine the level of resilience, the Connor and Davidson Resilience Scale (54) was used. This scale includes 25 items and is scored from 0 to 5 (totally false to totally true). The maximum score is 100 and the minimum score is 0. Cronbach’s alpha for the full scale was 0.89. Also, this scale scores were positively correlated with the Kobasa hardiness measure (r =0.83). Test–retest reliability showed a high level of agreement, with an intraclass correlation coefficient of 0.87. The Cronbach’s alpha coefficient of the Persian version was 0.77 (55). In this study, the Cronbach’s alpha for this scale was 0.73.

***The Positive and Negative Affect Scale***

The positive and negative affect scale is a self-report inventory that includes 20 items and was developed by Watson, Clark and Tellegen (56). This scale is scored based on a Likert-type scale, ranging from 1 (very low) to 5 (very high). The first 10 items assess positive affect and the second 10 examine negative affect. High scores indicate high levels of positive and negative affect. The Cronbach’s alpha coefficients were 0.88 for positive affect and 87.0 for negative affect. In addition, the test-retest reliability conducted with an interval of 8 weeks indicated that the coefficients of positive and negative affect were 0.68 and 0.71, respectively. Also, the relationship between Beck Depression Inventory with positive and negative affect was -0.35 and 0.51 (56). The Cronbach’s alpha coefficient of the Persian version was 0.87 (46). In this study, the alpha coefficient of this scale was found to be 0.82. 

***Data Analyses***

In the present study, to examine the descriptive characteristics of the variables, mean (sd) were applied. Moreover, path analysis was conducted to examine the mediating role of the variables. The obtained data were analyzed using SPSS22 and AMOS22. In path analysis, to perform the model fit indices, the chi-square goodness-of-fit test (chi-square/df), root mean square error of approximation (RMSEA), Goodness-of-Fit Index (GFI), Adjusted Goodness of Fit Index (AGFI), Comparative Fit Index (CFI), and Normed Fit Index (NFI) were calculated. Also, to assess the mediating role, the bootstrap method was applied using the macro of Preacher and Hayes (57). 

***Ethical Consideration***

This study (CT-72920701921001) was approved by Azad University of Zahedan. The basic objectives of the study were stated to the participants, and they were assured of the confidentiality of the obtained information and Informed consents were received.

## Results

The mean (sd) age of the participants was 15.06 (1.14) and the age range was 14 to 17 years old. The participants’ distribution across gender is close to equal (42% girls vs. 58% boys). At first, the parametric tests’ assumptions were checked. The main tests for the assessment of normality are Kolmogorov-Smirnov. Results (Table 1) showed the data distribution is normal.

One of these assumptions is that the observations are independent. The Durbin-Watson statistic is 2.157 which is between 1.5 and 2.5 and therefore the data is not autocorrelated.

The descriptive statistics and the matrix of the Pearson correlation coefficients of all the variables are presented in Table 2.

The mediating role of positive and negative affect in the association between perceived parenting styles and resilience was examined. The standard coefficients and significant indicators showed that the hypothetical model was not significant, such that negative affect was not correlated with any of the variables. Moreover, the perceived parenting of involvement was not correlated with positive affect and resilience. Therefore, the considered insignificant relationships were excluded from the model. Given the fit indices (X2/df =1.19, RMSEA = 0.055, GFI = 0.985, NFI = 0.977, CFI = 0.996), the final model was highly significant. In addition, the perceived parenting styles predicted 33% of the variance in positive affect and these 2 variables together predicted 54% of the variance in resilience. Figure 2 illustrates the standard coefficients of the variables in the final model. 

According to standardized path coefficients, parental warmth had a direct effect on positive affect (β = 0.31, P < 0.01), and resilience (β = 0.24, P < 0.05). Also, parental autonomy support had a direct effect on positive affect (β = 0.35, P < 0.01) and resilience (β = 0.23, P < 0.05). Furthermore, positive affect had a direct effect on resilience (β = 0.44, P < 0.001). Parental autonomy support and parental warmth could indirectly influence resilience (β = 0.29) with the help of the mediating role of positive affect.

To examine the mediating role, the bootstrap method was applied using the macro of Preacher and Hayes (57), the confidence interval of the studied path was 95% and the number of 1000 bootstrap resamples was obtained from 0.0531 (lower limit) to 0.0980 (upper limit). Therefore, since 0 was not between the lower limit and the upper limit, it can be noted that positive affect plays a medicating role in the relationship between perceived parenting styles and resilience.

**Table 1:** Kolmogorov-Smirnov Test of Perceived Parenting Styles, Affects, and Resilience

**Variables**	**Statistic**	**Sig**	**Skewness**	**Kurtosis**
Parental involvement	0.085	0.200	-0.147	-0.964
Parental autonomy support	0.114	0.055	-0.253	-0.954
Parental warmth	0.099	0.068	0.774	1.066
Positive affect	0.084	0.200	-0.323	-0.556
Negative affect	0.109	0.062	-0.020	-0.543
Resilience	0.079	0.200	0.254	0.302

**Table 2:** The Descriptive Statistics and the Correlation Matrix of Perceived Parenting Styles, Affects, and Resilience

**Variables**	**1**	**2**	**3**	**4**	**5**	**6**	**SD**	**Mean**
1- Parental involvement	1						8.56	38.49
2- Parental autonomy support	0.41**	1					10.95	74.22
3- Parental warmth	0.46**	0.38**	1				12.82	48.97
4- Positive affect	0.43**	0.45**	0.47**	1			4.36	34.90
5- Negative affect	0.13	0.11	0.07	-0.05	1		4.62	35.82
6- Resilience	0.41**	0.52**	0.54**	0.66**	0.22	1	10.79	85.38

Note. *P<0.05,

** P<0.01

**Figure 1:**
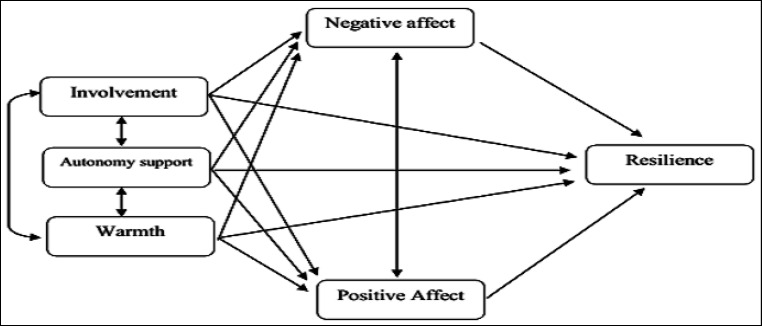
The Hypothetical Model of the Mediating Role of Positive and Negative Affect in the Association of Perceptions of Parenting Styles with Resilience

**Figure 2:**
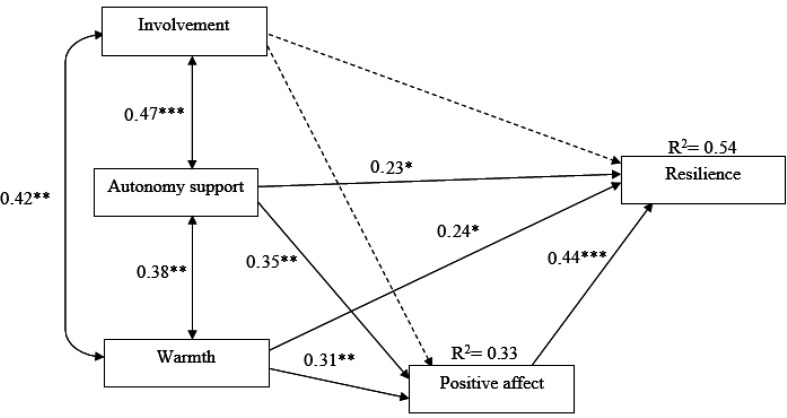
The Impact of the Perceived Parenting Styles in the Association of Perceptions of Parenting Styles with Resilience

## Discussion

The present study aimed to examine the mediating role of positive and negative affect in the association of the perceived parenting of involvement, autonomy support, and warmth with resilience among adolescents with addicted parents. Given the results obtained from this study, the direct effect of the perceived parenting styles on resilience was confirmed. The results indicated that perceived parenting styles were significantly and positively related to resilience. This finding is in line with the results obtained from several studies (18, 19, 21, 22, 24, 26). Crawford and Novak (25), who examined the positive and negative outcomes of perceived parenting styles, demonstrated that parenting styles led to an increase in resilience among adolescents. The findings of this study are also consistent with those of some previous studies (16, 20). 

Mediated by positive affect, perceived parenting styles indirectly affected resilience. To determine the mediating role of positive affect, the relationships of the perceived parenting styles with positive affect can be mentioned. Research showed that perceived parenting styles were related to happiness (43), high self-esteem, and hopefulness (28, 29), high life satisfaction (30, 31), high levels of well-being (32), low depression (33), proper coping styles and strategies (34, 35), and development of emotion regulation in children (41, 42). On the other side, useful emotion regulation strategies were significantly related to positive affect (49). Moreover, high self-confidence promoted the level of happiness among people (43). Therefore, this results are in line with those of other studies conducted to examine the relationship of the perceived parenting styles with positive affect. To explain the indirect effect of perceived parenting styles, the relationship between positive affect and resilience should be mentioned. One of the most important sources of resilience is positive affect (45, 50, 51). Research has shown that resilience was significantly associated with optimism (47, 48). Furthermore, since positive emotions increase mental flexibility and creativity, they play a significant role in enhancing resilience (44). Overall, the results showed that positive affect is one of the mechanisms through applying which parenting styles can be effective in promoting resilience among adolescents with addicted parents. In this regard, positive affect which results from the perceived parenting of autonomy support and warmth can improve resilience among adolescents.

Negative affect was not related to any of the variables. As a result, the indirect effect of perceived parenting styles on resilience mediated by negative affect was not significant. This findings are not consistent with those of other studies (51). To explain this finding, factors affecting the parenting styles mentioned in some studies should be noted. Several studies have pointed out that perceived parenting styles may vary in different cultures, and thus be associated with different outcomes (36-38). Moreover, participants’ age can be considered as another factor. Among the participants who were in the middle or late adolescence, the negative effects of low perceived parenting of involvement, autonomy support, and warmth were neutralized by other factors, including the effect of the school (27). This is why low perceived parenting of involvement, autonomy support, and warmth may not lead to negative affect. The results of a study conducted by Heaven and Ciarrochi (28) showed that the authoritarian and permissive parenting styles were not related to low self-esteem among adolescents. Moreover, when examining the impacts of parenting styles on children, the role of genetic and biological factors should be considered (33). Considering the mentioned reasons, the fact that negative affect was not related to the variables of this study can be explained.

## Limitation

The fact that this was a cross sectional study and the questionnaires were used to collect the required data can be mentioned as the main limitations of this study. In this regard, the results may be influenced by personal biases that may occur in self-report studies. Moreover, the method of sampling was accessible sampling.

## Conclusion

Based on the obtained results, perceived parenting styles can directly and indirectly predict resilience. Therefore, through promoting positive affect, adolescents’ perceived parenting styles can enhance resilience among adolescents. 
